# Characteristics and risk assessment of potentially toxic elements pollution in river water and sediment in typical gold mining areas of Northwest China

**DOI:** 10.1038/s41598-024-63723-3

**Published:** 2024-06-03

**Authors:** Yuhu Luo, Na Wang, Zhe Liu, Yingying Sun, Nan Lu

**Affiliations:** 1 Shaanxi Provincial Land Engineering Construction Group Co., Ltd., Xi’an, China; 2 Institute of Land Engineering and Technology, Shaanxi Provincial Land Engineering Construction Group Co., Ltd., Xi’an, China; 3 Key Laboratory of Degraded and Unused Land Consolidation Engineering, Ministry of Natural Resources, Xi’an, China

**Keywords:** Potential toxic elements, Water, Sediments, Environmental risk, Environmental monitoring, Pollution remediation

## Abstract

To assess the concentration characteristics and ecological risks of potential toxic elements (PTEs) in water and sediment, 17 water samples and 17 sediment samples were collected in the Xiyu River to analyze the content of Cr, Ni, As, Cu, Zn, Pb, Cd and Hg, and the environmental risks of PTEs was evaluated by single-factor pollution index, Nemerow comprehensive pollution index, potential ecological risk, and human health risk assessment. The results indicated that Hg in water and Pb, Cu, Cd in sediments exceeded the corresponding environmental quality standards. In the gold mining factories distribution river section (X8-X10), there was a significant increase in PTEs in water and sediments, indicating that the arbitrary discharge of tailings during gold mining flotation is the main cause of PTEs pollution. The increase in PTEs concentration at the end of the Xiyu River may be related to the increased sedimentation rate, caused by the slowing of the riverbed, and the active chemical reactions at the estuary. The single-factor pollution index and Nemerow pollution index indicated that the river water was severely polluted by Hg. Potential ecological risk index indicated that the risk of Hg in sediments was extremely high, the risk of Cd was high, and the risk of Pb and Cu was moderate. The human health risk assessment indicated that As in water at point X10 and Hg in water at point X9 may pose non-carcinogenic risk to children through ingestion, and As at X8–X10 and Cd at X14 may pose carcinogenic risk to adults through ingestion. The average HQ_ingestion_ value of Pb in sediments was 1.96, indicating that the ingestion of the sediments may poses a non-carcinogenic risk to children, As in the sediments at X8–X10 and X15–X17 may pose non-carcinogenic risk to children through ingestion.

## Introduction

Potentially toxic elements (PTEs) contamination is considered one of the most prominent issues affecting water quality^1^. Most rivers in the world have been affected by varying degrees of PTEs pollution, and the pollution is increasing year by year^2,3^. PTEs entering rivers are easily stored in sediments. When the environmental conditions change, PTEs in sediments will be released into the water. Due to its high toxicity, resistance to decomposition, and bioaccumulation, it poses significant environmental risks to river ecosystems^4–7^. Moreover, PTEs pollution can enter the human body through drinking water or the food chain, posing a threat to human health^8,9^. Research has shown that a small amount of PTEs can lead to various cardiovascular diseases, respiratory diseases, and even cancer^10–12^.

The sources of PTEs in rivers include surface runoff, emissions from industrial and mining activities, and atmospheric deposition^13^. Rivers flowing through mining areas are more susceptible to the threat of tailings and mining waste discharge^14,15^. Tailings exposed to the air for a long time are prone to releasing PTEs into rivers through weathering, posing a threat to the ecological health of rivers. The PTEs contamination incidents caused by mining activities have frequently occurred, such as the indiscriminate discharge of tailings in the Dabaoshan Mining area has caused serious PTEs pollution in the Henghe River^16^, and the mining of Dexing copper mine has led to severe pollution of Jishui River, and the PTEs has spread to farmland soil through river irrigation^17^.

**Figure 1:**
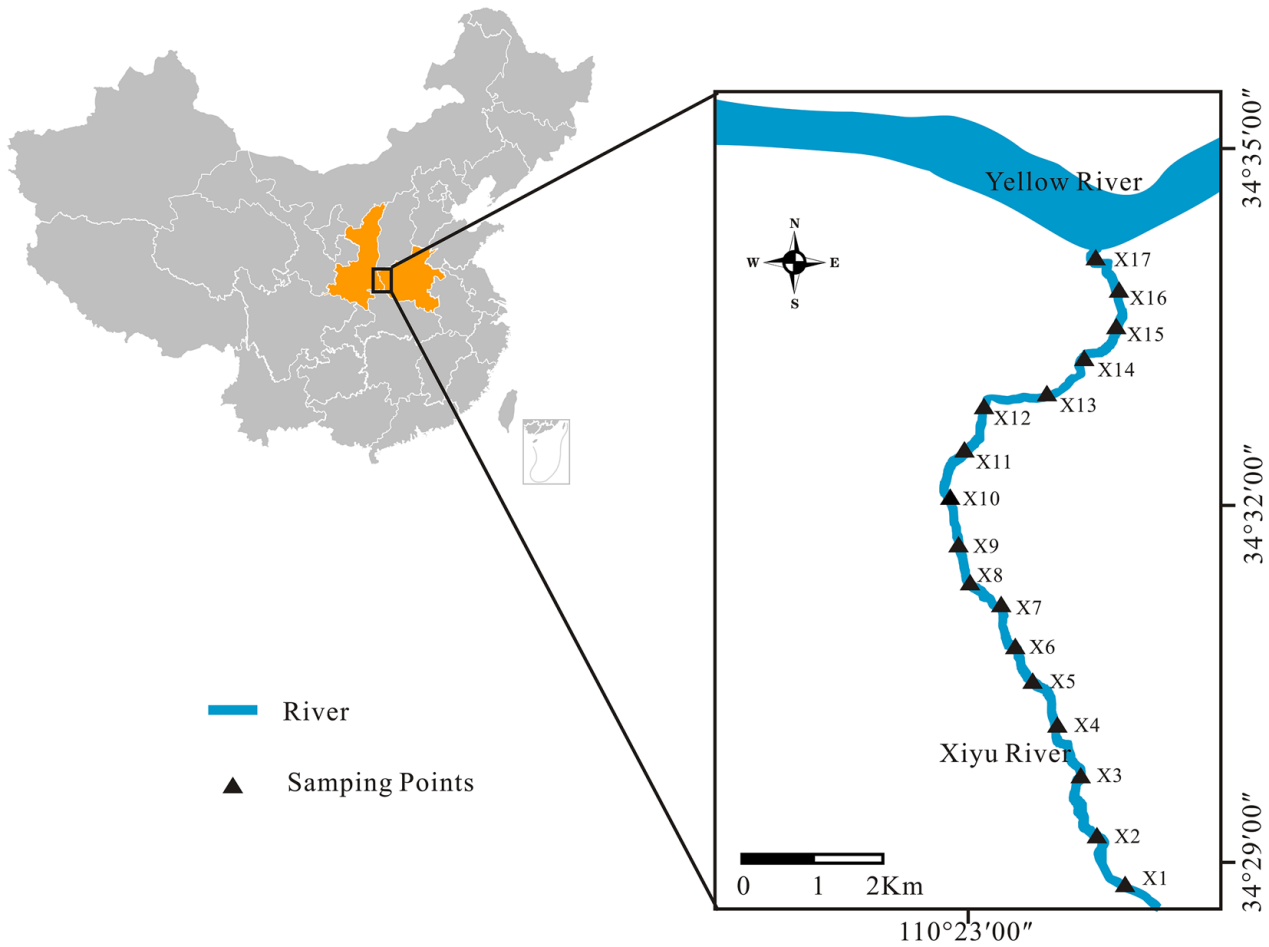
Location of the study area and the distribution of sampling points.

The gold mining in the research area can be traced back to 1104 of the Northern Song Dynasty, and large-scale mining began in 1975. The X8–X10 reaches of the Xiyu River is the main flotation area for gold mines (Fig. 1). In the last century, there was a period of disorderly mining, with many small artisanal Au mining flotation factories distributed on both sides of the river. These factories discharge the tailings, without any treatment, into the river channel^18^. It was not until the government issued a ban in 1996 that these small artisanal Au mining plants were significantly reduced, and the arbitrary discharge of tailings was curbed. However, the pollution caused by long-term disorderly mining to the environment of the mining area often persisted for a long time^19,20^. The present study collected the water samples and the sediment samples from the Xiyu River and measured the concentration of Cr, Ni, As, Cu, Zn, Pb, Cd, and Hg in the water and sediment. A comprehensive evaluation of PTEs pollution in the river was evaluated by single-factor pollution index, Nemerow comprehensive pollution index, potential ecological risk, and human health risk assessment. The objective of this study is to investigate the content and distribution characteristics of PTEs in the water and sediment of the Xiyu River, and to evaluate the ecological risk of PTEs pollution in the river. The results of the study will provide guidance for the protection of water systems in the Xiaoqinling mining area.

## Materials and methods

### Study area

The Xiaoqinling gold mine is the second largest gold production area in China, mainly developing quartz vein type gold ores, accompanied by pyrite, galena, sphalerite, chalcopyrite, magnetite, etc.^21^. The research area is in northwest China (110°20′00′′ E–110°27′00′′ E, 34°25′00′′ N–34°34′00′′ N). It has a typical temperate zone terrestrial monsoon arid climate. The annual average temperature is 13.15 ℃, and the annual average precipitation is 623.8 mm.

The Xiyu River originates from the ridge of Qinling Mountains, flows through the Xiaoqinling Mining Area, and flows into the Yellow River in the northwest of Lingbao City. The river is about 15 km long and has a river basin area of 28 square kilometers. The sediment thickness of the river is generally 10–30 cm, with a greenish gray color and relatively fine particles. Except for the X8–X10 reaches, which is the main flotation area for gold mining, the other areas are mainly engaged in agricultural production activities (Fig. 1). Because Xiyu River is the boundary river between Shaanxi and Henan provinces, there are gold mining plants distributed on both sides of the river, making it one of the most severely polluted rivers in Xiaoqinling gold-mining region.

### Sample collection and analysis

Seventeen sampling points were arranged with a spacing of approximately 1000 m from the upstream to downstream of the river (Fig. 1), collecting the surface water samples and the sediment samples at each point. The water sample were filtered through 0.45 μm glass fiber filter membranes immediately after collection to remove large suspended solids. Next, HNO_3_ was added to ensure the pH was less than 2, and then the samples were kept sealed at 4 °C^22^. Three adjacent locations were selected near each sampling point, and approximately 10 cm thick sediment was collected at each location. Then these three sediment samples were mixed into one as the final sample for each sampling point, and quickly stored the samples in a 4 ℃ refrigerator. The sediment samples used for PTEs determination were dried in the dark and ground to 100 mesh using an agate mortar. The concentrations of Cr, Ni, As, Cu, Zn, Pb, Cd in samples were determined by inductively coupled plasma mass spectrometer system (ICP-MAS, PE300D)^23^, and Hg was determined by atomic fluorescence spectrometer (AFS-9760)^24^. The recovery rates of PTEs contents in standard reference materials were between 90 and 110%.

### Environmental risk assessment

#### Pollution index

Single-factor pollution index and Nemerow pollution index are often used to evaluate the pollution status of PTEs in water^25,26^. The single-factor pollution index was used to evaluate the pollution level of single PTE in water, and was calculated as shown in following equation:

1 $${\text{P}}_{{\text{i}}} {\text{ = C}}_{{\text{i}}} {\text{/B}}_{{\text{i}}}$$

where, P_i_ represents the single-factor pollution index of element i, C_i_ represents the actual concentration of i (mg L^−1^), and B_i_ represents the evaluation standard of i. In present study the surface water environmental quality standard of the National Environmental Protection Agency of China (GB 3838-2002) was used as the evaluation standard^27^.

Nemerow pollution index not only reflects the pollution degree of single PTE, but also describes the comprehensive pollution of multiple PTEs. Additionally, it highlights the impact and effect of the pollutant with the largest pollution index on environmental quality. It is a comprehensive method widely used for evaluating water environmental quality at present. The Nemerow pollution index was calculated as shown in Eq. (2)^28^.

2 $${\text{P}}_{{\text{n}}} = \sqrt {0.5 \times \left[ {\max \left( {{\text{P}}_{{\text{i}}} } \right)^{2} + {\text{ave}}({\text{P}}_{{\text{i}}} {)}^{2} } \right]}$$

where, P_i_ represents the single-factor pollution index of i; max (P_i_) represents the maximum value of P_i_, and ave (P_i_) represents the average value of P_i_. The classifications of P_i_ and P_n_ are shown in Table 1^29,30^.

**Table 1:** Classification of pollution levels of pi and P_n_.

P_i_	Pollution level	P_n_	Pollution degree
P_i_ < 1	Unpolluted	P_n_ ≤ 0.7	Safe
1 ≤ P_i_ < 2	Slightly polluted	0.7 < P_n_ ≤ 1	Precaution
2 ≤ P_i_ < 3	Moderately polluted	1 < P_n_ ≤ 2	Slight pollution
3 ≤ P_i_ < 5	Highly polluted	2 < P_n_ ≤ 3	Moderate pollution
P_i_ ≥ 5	Very highly polluted	P_n_ > 3	Heavy pollution

#### potential ecological risks

The potential ecological risk index was proposed by Hakanson in 1980^31^. This method evaluates PTEs pollution in soils or sediments from the perspective of sedimentology according to the nature of PTEs and environmental behavior characteristics. While considering the content of PTEs in the soil, this method links the ecological and environmental effects with toxicology and can more accurately represent the impact of PTEs on the ecological environment. The expressions are shown in the following equations:

3 $${\text{C}}_{{\text{f}}}^{{\text{i}}} { = }\frac{{{\text{C}}_{{\text{s}}}^{{\text{i}}} }}{{{\text{C}}_{{\text{n}}}^{{\text{i}}} }}$$

4 $${\text{E}}_{{\text{r}}}^{{\text{i}}} \; = \;{\text{T}}_{{\text{r}}}^{{\text{i}}} \times {\text{C}}_{{\text{f}}}^{{\text{i}}}$$

5 $${\text{RI}}\;{ = }\;\mathop \sum \limits_{{\text{i = 1}}}^{{\text{n}}} {\text{E}}_{{\text{r}}}^{{\text{i}}}$$

where, ${\text{C}}_{{\text{f}}}^{{\text{i}}}$ is the pollution coefficient of element i, ${\text{C}}_{{\text{s}}}^{{\text{i}}}$ is the measured concentration value of i in sediment, mg kg^−1^; ${\text{C}}_{{\text{n}}}^{{\text{i}}}$ is the background value of i, derived from the element concentration in the nearby river sediments that are almost unaffected by human activities^32^; ${\text{E}}_{{\text{r}}}^{{\text{i}}}$ is the potential ecological risk index of i; ${\text{T}}_{{\text{r}}}^{{\text{i}}}$ is the toxicity response parameter of i. Specifically, the toxic response coefficient of PTEs is Hg = 40, Cr = 2, Cd = 30, As = 10, Pb = 5, Cu = 5, Zn = 1, Ni = 5^33^; RI is the potential ecological risk index for multiple PTEs. The classification of potential ecological risk index is shown in Table 2.

**Table 2:** Classification of potential ecological risk index.

Ecological risk level	Low	Moderate	Considerable	High	Significantly high
${\text{E}}_{\text{r}}^{\text{i}}$	< 40	40–80	80–160	160–320	> 320
RI	< 150	150–300	300–600	≥ 600	/

#### Human health risk assessment

PETs can enter the human body through ingestion, skin absorption and respiration. For humans, ingestion and skin absorption are the two main exposure pathways of PTEs in water and sediments^34,35^. The daily dose of PTEs entering the human body through ingestion and skin absorption, and the carcinogenic risk and non-carcinogenic risk of PTEs to the human body were determined according to relevant documents from the US Environmental Protection Agency^36^, using the following equations^37,38^:

6 $${\text{ADD}}_{{{\text{ingestion}}}} {\text{ - sediment}} = \frac{{{\text{C}}_{{\text{s}}} \times {\text{I}}_{{\text{s}}} \times {\text{EF}} \times {\text{ED}}}}{{{\text{BW}} \times {\text{AT}}}} \times 10^{ - 6}$$

7 $${\text{ADD}}_{{{\text{dermal}}}} {\text{ - sediment}}\; = \;\frac{{{\text{C}}_{{\text{s}}} \times {\text{SA}} \times {\text{SL}} \times {\text{ABF}} \times {\text{EF}} \times {\text{ED}}}}{{{\text{BW}} \times {\text{AT}}}} \times {\text{10}}^{{ - {\text{6}}}}$$

8 $${\text{ADD}}_{{{\text{ingestion}}}} {\text{ - water}} = \frac{{{\text{C}}_{{\text{w}}} \times {\text{I}}_{{\text{w}}} \times {\text{EF}} \times {\text{ED}}}}{{{\text{BW}} \times {\text{AT}}}}$$

9 $${\text{ADD}}_{{{\text{dermal}}}} {\text{ - water}} = \frac{{{\text{C}}_{{\text{w}}} \times {\text{SA}} \times {\text{K}}_{{\text{p}}} \times {\text{ET}} \times {\text{EF}} \times {\text{ED}}}}{{{\text{BW}} \times {\text{AT}}}} \times 10^{ - 3}$$

10 $${\text{HI}}\; = \;\sum {\text{HQ}}\; = \;\sum \frac{{{\text{ADD}}}}{{{\text{RfD}}}}$$

11 $${\text{TCR}}\; = \;\sum {\text{CR}}\; = \;\sum {\text{ADD}} \times {\text{SF}}$$

where ADD_ingestion_ and ADD_dermal_ are the daily dose of ingestion and skin absorption respectively, C_s_ is PTE content in sediment, C_w_ is PTE content in water, I_s_ is the daily intake of sediment, I_w_ is the average daily drinking water intake, EF is the exposure frequency, ED is the exposure time; BW is the average body weight, AT is the average exposure time; SA is the exposed area of skin; SL is skin adhesion factor; ABF is a skin adsorption factor. HQ is the hazard quotient and HI is the hazard index. The specific exposure parameters are shown in Table 3, and the RfD, CSF and K_p_ values of PTEs are shown in Table 4^39,40^. HQ and HI are used to describe the non-carcinogenic risks of PTEs. When HQ or HI < 1, it indicates no non-carcinogenic health risks; On the contrary, it indicates the existence of potential non-carcinogenic health risks, and the higher the value, the higher the risk. CR and TCR are used to describe the carcinogenic risk of PTEs, when CR is less than 10^–6^, it indicates no carcinogenic risk. When CR is between 10^–6^ and 10^–4^, it indicates an acceptable risk range, and when CR is greater than 10^–4^, it indicates that PTEs in water or sediment are highly likely to pose a carcinogenic risk to humans^36^.

**Table 3:** Exposure parameters for the health risk assessment models.

Parameters	Unit	Value	
		Child	Adult
I_s_	mg d^−1^	200	100
I_w_	L d^−1^	0.64	2
EF	d year^−1^	350	350
ED	years	6	30
BW	kg	15	70
AT	d	2190 (For non-carcinogens)	10,950 (For non-carcinogens)
		25,550 (For carcinogens)	25,550 (For carcinogens)
SA	cm^2^	6600	18,000
SL	mg cm^−2^	0.2	0.07
ABF	/	0.001 (For non-carcinogens)	0.001 (For non-carcinogens)
		0.01 (For carcinogens)	0.01 (For carcinogens)
ET	h	1	0.58

**Table 4:** RfD, SF and K_p_ of different exposure pathways of PTEs.

Element	RfD_ingestion_	RfD_dermal_	SF_ingestion_	SF_dermal_	K_p_ (cm h^−1^)
	(mg kg^−1^ day^−1^)				
Cr	3.00E−03	6.00E−05	–	–	0.002
Ni	2.00E−02	5.40E−03	–	–	0.0002
Cu	4.00E−02	1.20E−02	–	–	0.001
Zn	3.00E−01	6.00E−02	–	–	0.0006
As	3.00E−04	1.23E−04	1.50E+00	3.66E+00	0.001
Cd	1.00E−03	1.00E−05	6.10E+00	6.10E+00	0.001
Pb	3.50E−03	5.25E−04	8.50E−03	–	0.0001
Hg	3.00E−04	2.10E−05	–	–	0.001

## Results and discussion

### PTEs in sediment and water

**Figure 2:**
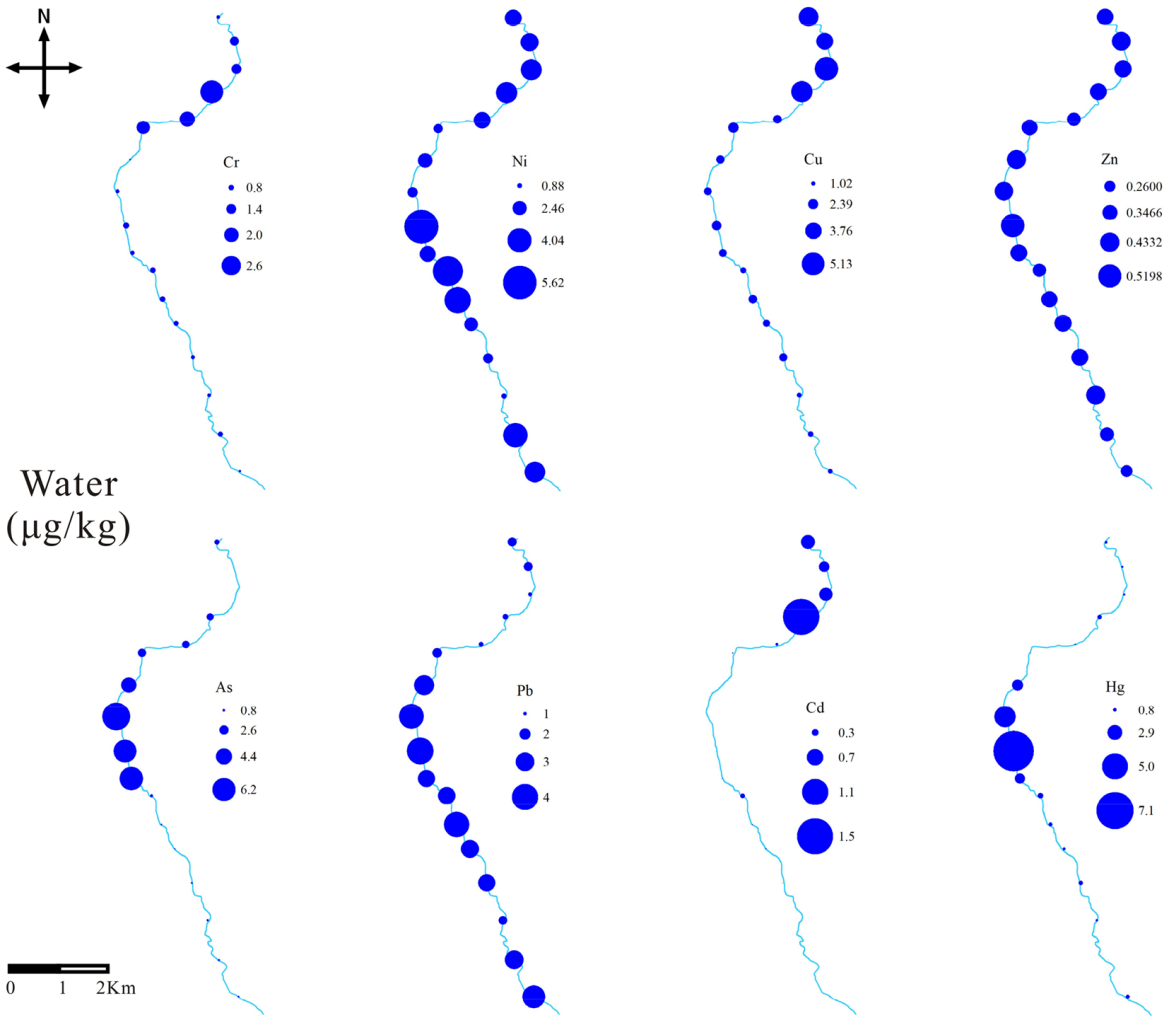
The distribution of PTEs in the water along Xiyu River.

Table 5 presents the statistical results of PTEs concentrations in sediments and water of the Xiyu River. The concentrations of PTEs in the water, from high to low, was Ni > Cu > As > Pb > Hg > Cr > Zn > Cd. Except for Hg, the concentrations of other elements in water were within the limits of the corresponding national standards (GB 3838-2002)^27^. The average concentration of Hg in water was 1.34, which exceeded the standard by 13.4 times. In X8–X10 reaches of the river, the concentration of Hg was abnormally high (Fig. 2), which exceeded the standard by 45.2 times. The high Hg concentration in this reaches may be related to the distribution of mining plants, which used the amalgamation technique for gold flotation, and a large amount of tailings with Hg were discharged into the river, causing Hg pollution^41^. In the downstream of the river, there was a significant decrease in Hg, which may be due to the self-purification of the river, where lots of Hg in water deposited into the sediment^42^. In the reaches with gold mining plants distribution (X8–X10), the concentrations of Hg, As, Ni, and Cd in the water were the highest, indicating that these elements in the river may be related to the discharge of tailings. The concentration of PTEs in the water of the estuary reaches (X15–X17) showed an increasing trend, which may be related to the active chemical reactions in the estuary^43^. Compared with the river water worldwide (Table 6), the concentration of Hg was lower than that of the Tano River in Ghana and higher than that of other rivers. Cu, Zn, Pb, Cd were smaller than most rivers such as Tarapaya, Ganga, Khoshk, etc. This is due to the government's strong control over disorder gold mining activities.

**Table 5:** PTEs contents in sediments and water of the Xiyu River.

Items	Water (μg kg^−1^)			Sediment (mg kg^−1^)		
	Range	Mean	GB 3838-2002	Range	Mean	GB 15618-2018
Cr	0.22–3.01	0.95 ± 0.71	50	66.98–125.39	90.95 ± 14.86	250
Ni	0.9–5.61	3 ± 1.21	20	32.75–113.33	58.6 ± 20.32	190
Cu	1.03–5.13	2.31 ± 1.26	1000	35.24–429.78	270.35 ± 140.47	100
Zn	0.26–0.52	0.37 ± 0.06	1000	68.61–337.78	162.53 ± 72.54	300
As	0.08–7.34	2.04 ± 2.29	50	5.47–37.72	17.75 ± 11.11	25
Cd	0.01–1.5	0.22 ± 0.37	5	0.16–12.1	1.81 ± 3.02	0.6
Pb	0.54–3.53	2.02 ± 0.97	50	28.3–1567.82	537.27 ± 395.54	170
Hg	0.07–7.59	1.34 ± 1.82	0.10	0.27–9.16	2.69 ± 2.17	3.4

**Table 6:** Comparison of PTEs in the water and sediment of the Xiyu River with world’s rivers.

River		Cr	Ni	Cu	Zn	As	Cd	Pb	Hg
Present study	Water	0.95	3	2.31	0.37	2.04	0.22	2.02	1.34
	Sediments	90.95	58.6	270.35	162.53	17.75	1.81	537.27	2.69
Yangtze River^44^	Water	30	/	6950	22,720	190	260	n.d.	0.18
	Sediments	0.25	/	315.76	334.53	61.55	3.51	46.46	0.0239
Taipu River^45^	Water	3	0.2	6	7	0.5	0.9	2	/
	Sediments	80.58	36.93	48.81	144.22	13.15	0.32	38.24	/
Yellow River^46^	Water	0.08	1.89	1.78	1.79	2.37	< 0.01	0.01	ND
	Sediments	76.59	27.99	19.66	75.19	9.38	0.18	20.26	0.02
Muchawka^47^	Water	/	800	700	17,600	/	50	9300	/
	Sediments	/	4.8	2.6	19.1	/	0.6	11.7	/
Khoshk^48^		190	80	30	1700	/	30	70	/
	Sediments	187.87	107.6	42.25	64.81	/	1.23	121.01	/
Ganga^49^	Water	725	/	125	/	153	59	163	/
	Sediments	57.3125	/	19.122	/	0.14175	2.989	11.9675	/
Tano^50^	Water	266	/	ND	149	12	50	381	45
	Sediments	25.4	/	3.42	21.1	1.78	1.3	4.67	1.24
Tarapaya^14^	Water	/	/	13	601	/	5	56	/
	Sediments	/	/	296	9058	/	107	902	/
Old Brahmaputra^51^	Water	10	440	120	10	/	1	110	1
	Sediments	6.6	12.8	6.2	52.7	/	0.48	7.6	0.001

Where ND not detected, the units of water are all μg L^−1^, the units of sediment are mg kg^−1^.

The concentrations of PTEs in the sediments, from high to low, was Pb > Cu > Zn > Cr > Ni > As > Hg > Cd. The average concentrations of Pb, Cu, and Cd exceeded the corresponding national environmental quality standards (GB 15,618-2018) by 3.16, 2.70, and 3.01 times, respectively^52^. This result can be attributed to the relatively high content of Pb, Cu, and Cd in the tailings of the Xiaoqinling mining area. The tailings are randomly discharged into the river, leading to the release of PTEs into the river^53,54^.

**Figure 3:**
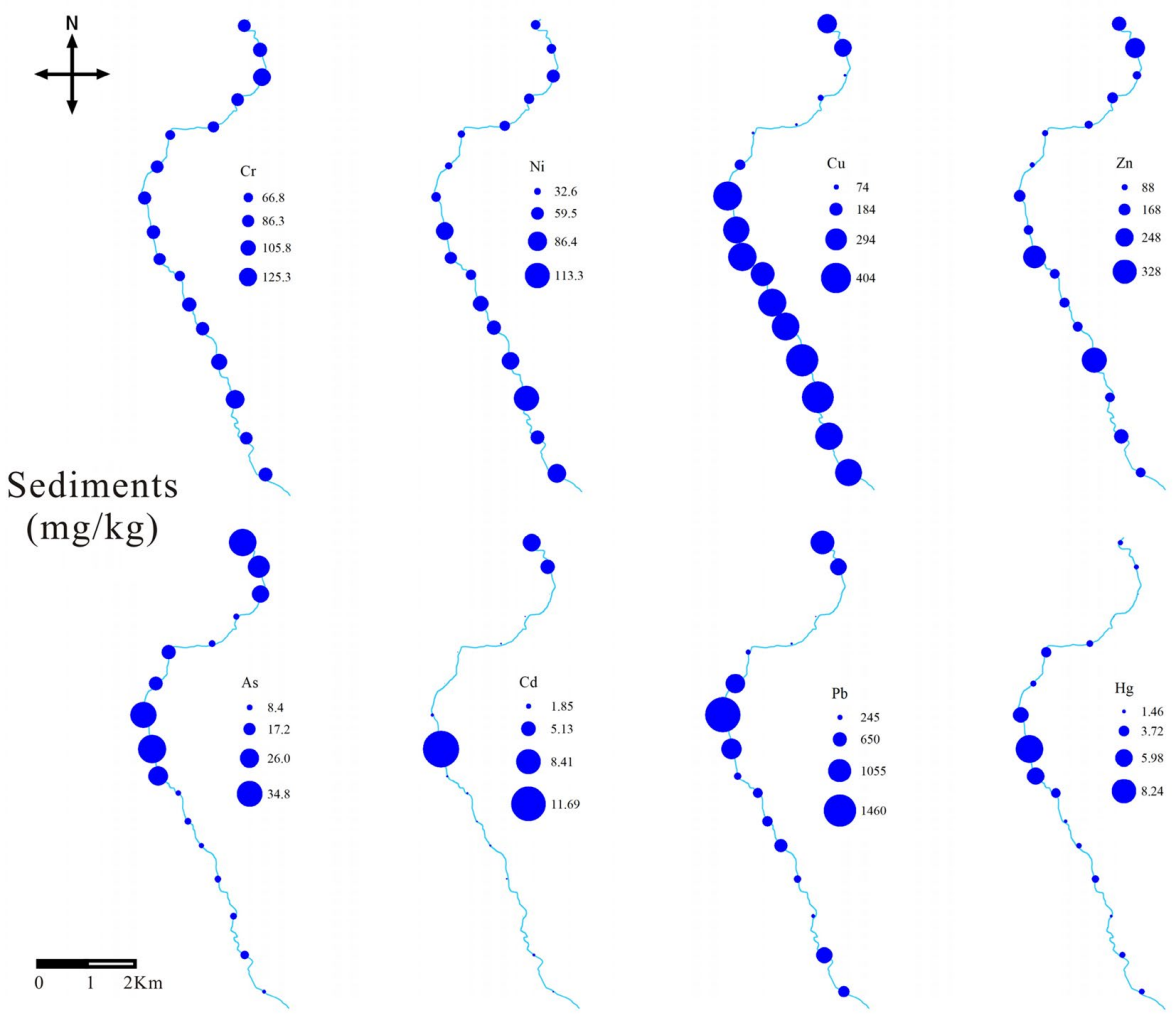
The distribution of PTEs in the sediments along Xiyu River.

Figure 3 shows the distribution characteristics of PTEs in the sediment of Xiyu River. Pb, Cu, Zn, Ni, As, Hg, and Cd showed a significant increasing trend in the X8–X10 reaches, indicating that these elements are related to the emissions from gold flotation plants. There was no significant change in Cr, which indicates that Cr may come from a background source related to rock weathering. At the estuary, there was a significant increase in the concentration of PTEs, which may be due to that the estuary area is in the Yellow River alluvial plain area, where the riverbed slows down and the rate of PTEs deposition increases^55^.

Compared with rivers around the world, the Hg in the sediment of the Xiyu River was higher than that of other rivers in Table 6. This result is like that of water in the Xiyu River, attributed to the discharge of tailings from gold flotation plants. Cu, Cr, and Cd in sediments were smaller than those in the Tarapaya River in the United States, which was polluted by tailings, but larger than most of the rivers in Table 6.

### Risk assessment of PTEs in water

#### Single-factor pollution index and Nemerow pollution index

**Figure 4:**
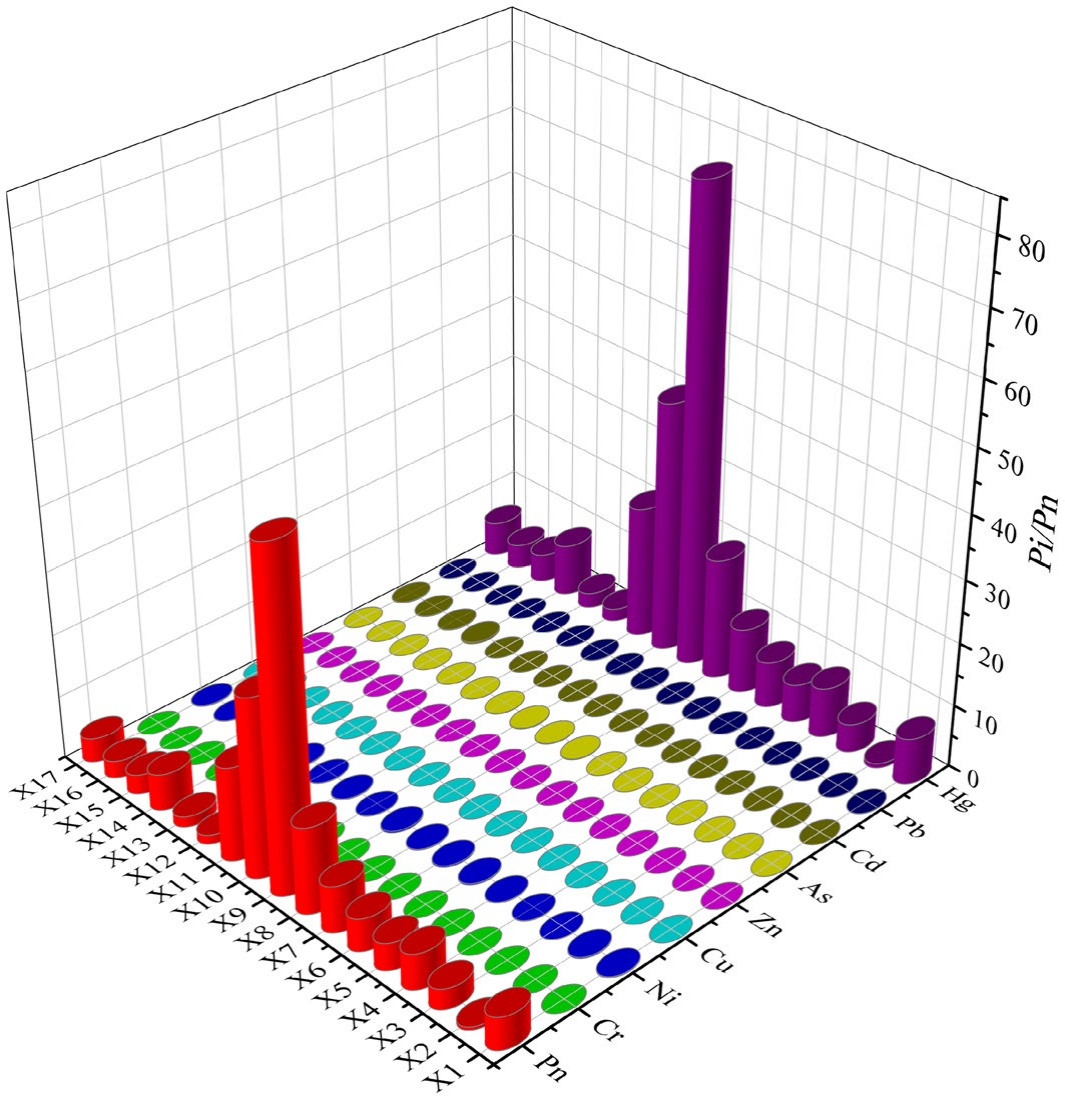
P_i_ and P_n_ of PTEs in the water of Xiyu River.

Figure 4 shows the single-factor pollution index and Nemerow pollution index of PTEs in water of the Xiyu River. Except for Hg, the Pi of other elements was less than 1, indicating an unpolluted state. The average Pi of Hg was 13.42, which represented extremely severe pollution, indicating that Hg is the main pollutant in the water. The average value of P_n_ in the Xiyu River was 9.5, indicating heavy pollution in PTEs. P_n_, as well as Pi of Hg, increased first and then decreased. In the X8-X10 reaches, the values of P_n_ and Pi of Hg were the highest, indicating that the main contribution of P_n_ comes from Hg.

#### Health risk assessment of PTEs in water

**Figure 5:**
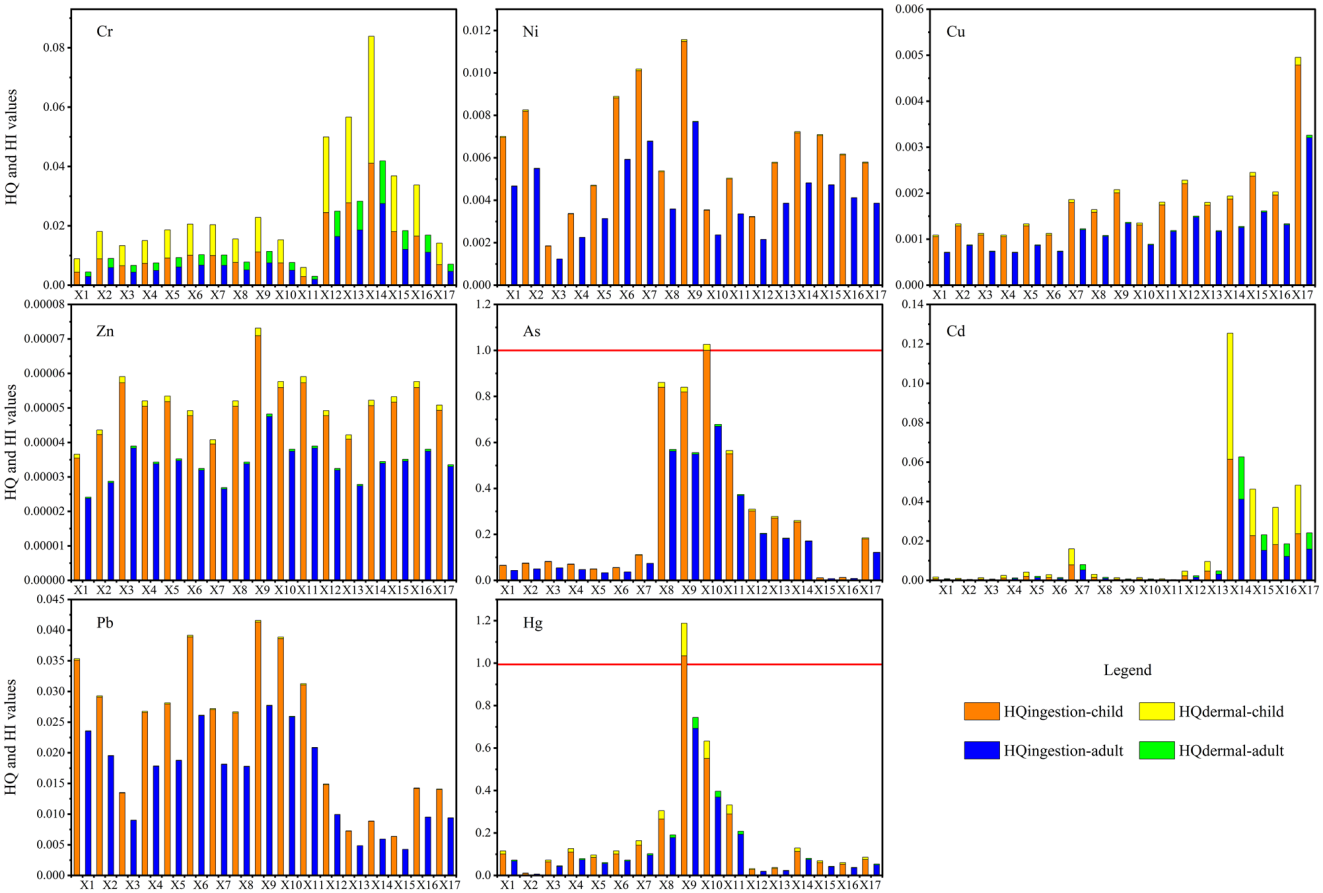
HQ and HI in the Xiyu River water.

Figure 5 shows the non-carcinogenic coefficients HQ and HI for children and adults caused by ingestion and skin contact of the Xiyu River water. The average HQ_ingestion_, HQ_dermal_, and HI of children and adults were all less than 1, while the HQ_ingestion_ of As for children was 1.001 at X10, the HQ_ingestion_ of Hg for children was 1.035 at X9, indicating that the intake of river water from these two reaches may pose a non-carcinogenic risk of As or Hg to children. HQ and HI for children were significantly higher than those for adults, indicating that PTEs in water are more toxic to children, and children should be kept away from polluted river to avoid poisoning caused by contact or ingestion of polluted river water.

**Figure 6:**
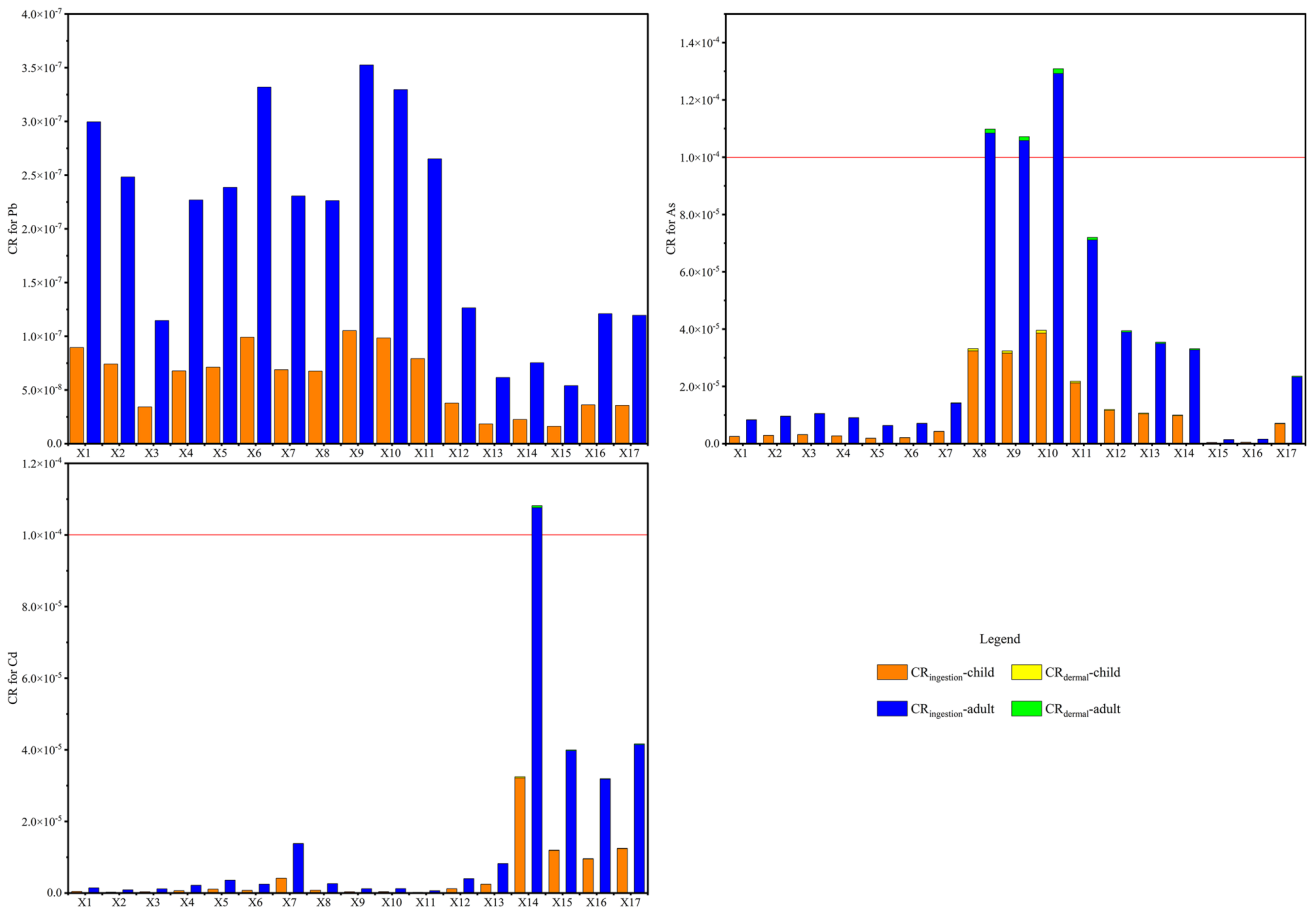
CR of the Xiyu River water.

Figure 6 shows the carcinogenic risk index CR. The average CR of As, Cd, and Pb for children and adults were within 10^–4^, the acceptable carcinogenic risk stated by the US Environmental Protection Agency. However, at the X8-X10 of the river, the CR of As for adults was 1.16 × 10^–4^, and at the X14 point, the CR of Cd for adults was 1.08 × 10^–4^, which are greater than 10^–4^ and may pose a certain As or Cd carcinogenic risk to adults. The CR of children is significantly lower than that of adults, which may be attributed to the larger skin area and more water intake of adults.

### Risk assessment of PTEs in sediments

#### Potential ecological risk assessment

**Figure 7:**
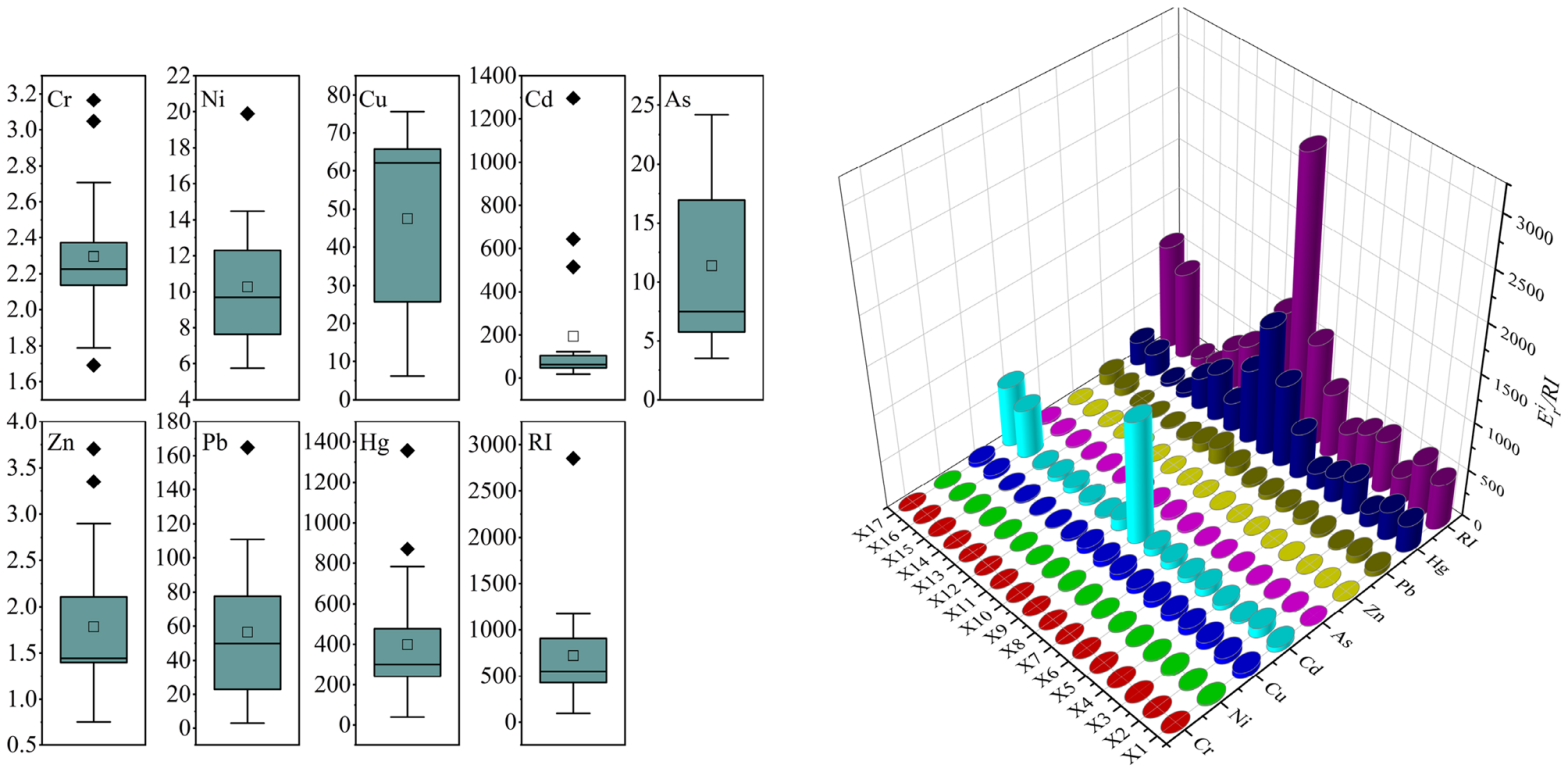
Potential ecological risk index of the Xiyu River Sediments.

Figure 7 shows the potential ecological risk index of PTEs in the sediment of the Xiyu River. The E_r_^i^ of Cr, Ni, As, and Zn were all less than 40, indicating a relatively low potential ecological risk. The average E_r_^i^ of Cu and Pb were 47.5 and 56.5, respectively, indicating moderate risk. The average E_r_^i^ of Cd was 193.7, indicating high risk, and the average E_r_^i^ of Hg was 398.4, indicating significantly high risk. The potential ecological risk index RI of the PTEs ranges from 97.44 to 2852.45, with significant changes. The average RI was 721.8, indicating high potential ecological risk. The RI value was the highest in the area (X8–X10) where the gold mining flotation factories were located, indicating that the potential ecological risk in the sediments of Xiyu River is mainly caused by gold mining activities.

#### Health risk assessment of PTEs in sediments

**Figure 8:**
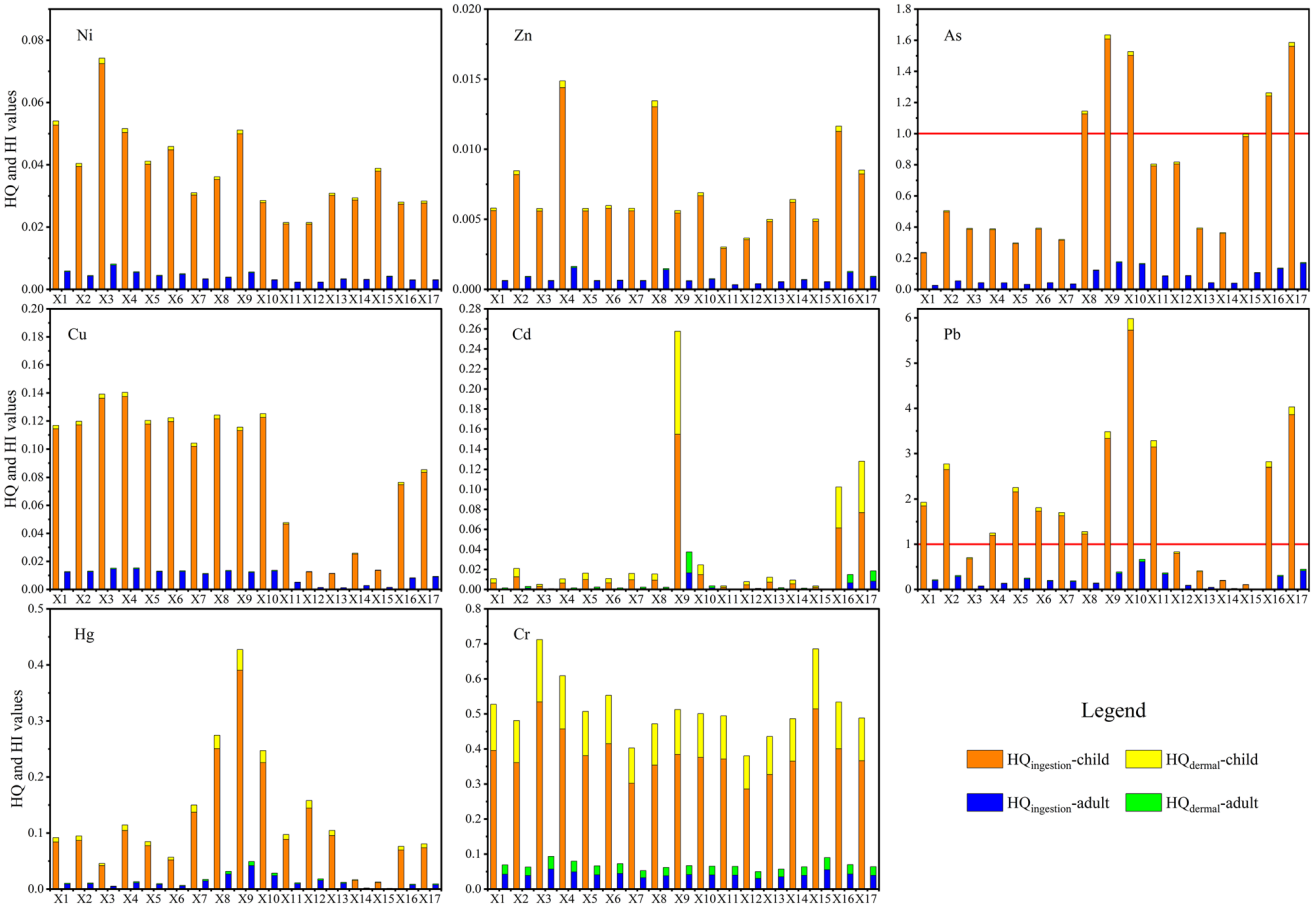
HQ and HI of the Xiyu River sediments.

Figure 8 shows the non-carcinogenic risk indices HQ and HI in the Xiyu River sediments. The results showed that HQ_ingestion_, HQ_dermal_, and HI of Cr, Ni, Cu, Zn, Cd, and Hg for children and adults were all less than 1, indicating that these PTEs may not pose non-carcinogenic risk to children and adults through ingestion and skin contact. The average HQ_ingestion_ of Pb was 1.96, indicating that the intake of sediments may poses a non-carcinogenic risk of Pb to children. The HQ_ingestion_ values of As were 1.41 and 1.26 in the factories distribution river section (X8–X10) and the estuary river section (X15–X17), respectively, which were higher than 1, indicating that the intake of sediments in these river sections may poses a non-carcinogenic risk to children. The non-carcinogenic risk of the PETs in water and sediments to children is higher than that to adults, which may be related to the daily behavior and physiological activities of children^56^.

**Figure 9:**
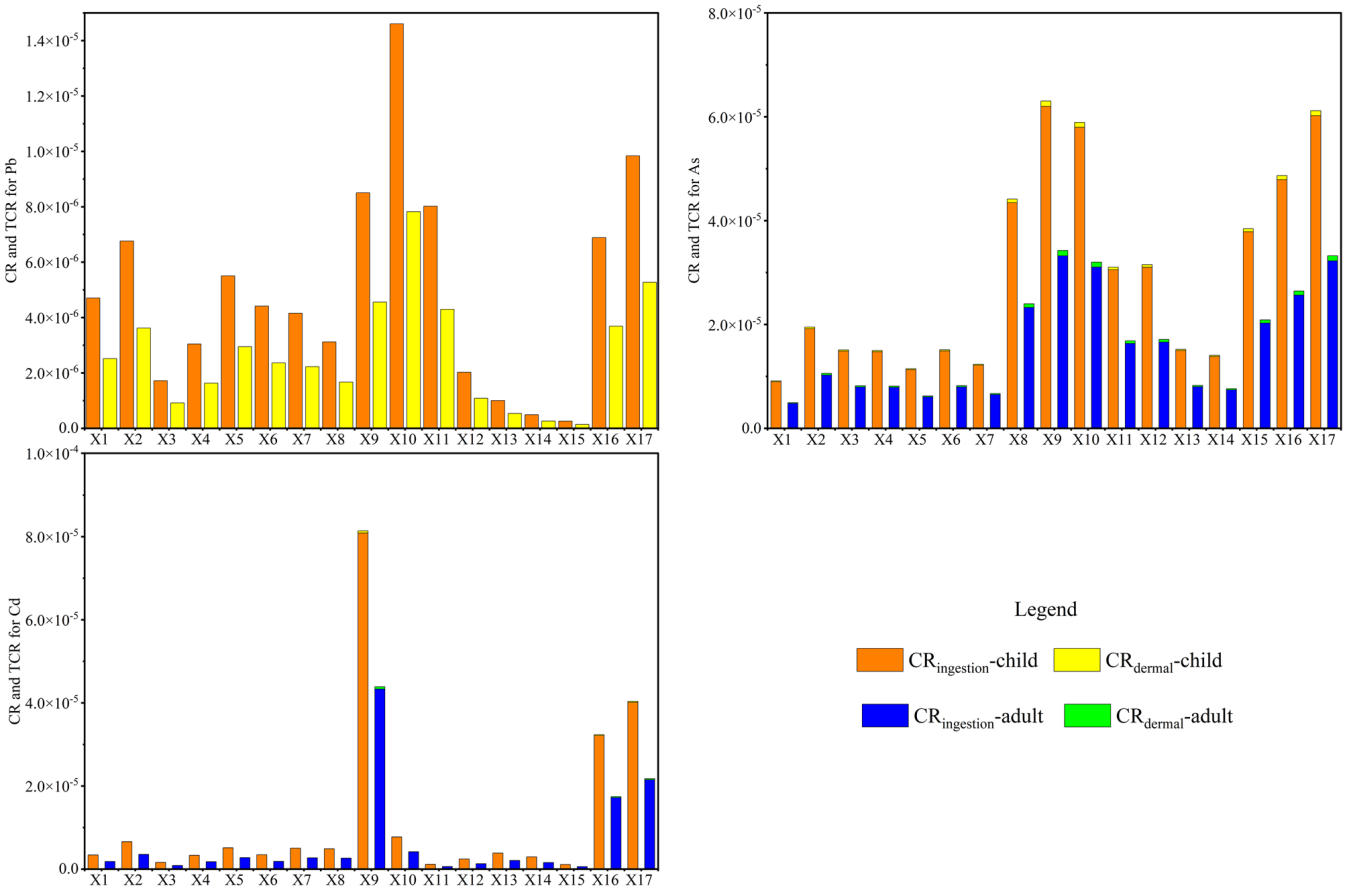
CR and TCR of the sediments in Xiyu River.

Figure 9 shows the carcinogenic risk index CR and TCR of PTEs in sediments of the Xiyu River. The result showed that the CR_ingestion_, CR_dermal_, and TCR of As, Cd, and Pb for children and adults were all less than 10^–4^, indicating that the carcinogenic risk is within an acceptable range. The CR_ingestion_ of children and adults was much higher than CR_dermal_, indicating that ingestion of sediments in the Xiyu River is the main pathway leading to cancer in children and adults.

## Conclusion

Monitoring and evaluating the river environment is of great significance for protecting the ecological environment of river basins. The present study system collected 17 sediment samples and 17 water samples from the Xiyu River and comprehensively evaluated the ecological risks of the river. The results indicated that the concentrations of PTEs in the water, from high to low, was Ni > Cu > As > Pb > Hg > Cr > Zn > Cd, and the concentrations of PTEs in the sediments, from high to low, was Pb > Cu > Zn > Cr > Ni > As > Hg > Cd. Due to long-term disordered gold mining activities, the Hg in the water exceeded the corresponding environmental quality standards, and the Pb, Cu, and Cd in the sediment exceeded the corresponding environmental quality standards. In the factories distribution river section (X8–X10), there was a significant increase in PTEs in water and sediments, indicating that the arbitrary discharge of tailings during gold mining flotation is the main cause of PTEs pollution in the Xiyu River. The increase in PTEs content at the end of the Xiyu River may be related to the increased sedimentation rate, caused by the slowing of the riverbed, and the active chemical reactions at the estuary.

The single-factor pollution index and Nemerow pollution index indicated that the river water was severely polluted by Hg. Potential ecological risk index indicated that the risk of Hg in sediments was extremely high, the risk of Cd was high, and the risk of Pb and Cu was moderate. The human health risk assessment indicated that As in water at point X10 and Hg in water at point X9 may pose a non-carcinogenic risk to children through ingestion, and As at X8–X10 and Cd at X14 may pose a carcinogenic risk to adults through ingestion. The average HQ_ingestion_ value of Pb in sediments was 1.96, indicating that the ingestion of the sediments may poses a non-carcinogenic risk to children, As in the sediments at X8–X10 and X15–X17 may pose a non-carcinogenic risk to children through ingestion. The non-carcinogenic risk of the PETs in the water and sediments to children was higher than that to adults, while the carcinogenic risk of the PETs in the water and sediments to children was lower than that to adults.

It is recommended to strengthen the control of disordered gold mining plants, avoid the arbitrary discharge of tailings during gold flotation, and take necessary treatment measures for river reaches with severe PTEs pollution.

## Data Availability

All data generated and analysed during this study are included in this published article.
